# Development and characterization of a novel luciferase based cytotoxicity assay

**DOI:** 10.1038/s41598-017-18606-1

**Published:** 2018-01-09

**Authors:** Hittu Matta, Ramakrishnan Gopalakrishnan, Sunju Choi, Rekha Prakash, Venkatesh Natarajan, Ruben Prins, Songjie Gong, Saurabh D. Chitnis, Michael Kahn, Xu Han, Vishan Chaudhary, Adam Soni, Jennifer Sernas, Prottasha Khan, Dan Wang, Preet M. Chaudhary

**Affiliations:** Jane Anne Nohl Division of Hematology and Center for the Study of Blood Diseases, University of Southern California, Keck School of Medicine, Los Angeles, California, United States of America

## Abstract

A simple, accurate, sensitive and robust assay that can rapidly and specifically measure the death of target cells would have applications in many areas of biomedicine and particularly for the development of novel cellular- and immune-therapeutics. In this study, we describe a novel cytotoxicity assay, termed the Matador assay, which takes advantage of the extreme brightness, stability and glow-like characteristics of recently discovered novel marine luciferases and their engineered derivatives. The assay involves expression of a luciferase of interest in target cells in a manner so that it is preferentially retained within the healthy cells but is either released from dead and dying cells or whose activity can be preferentially measured in dead and dying cells. We demonstrate that this assay is highly sensitive, specific, rapid, and can be performed in a single-step manner without the need for any expensive equipment. We further validate this assay by demonstrating its ability to detect cytotoxicity induced by several cellular and immune-therapeutic agents including antibodies, natural killer cells, chimeric antigen receptor expressing T cells and a bispecific T cell engager.

## Introduction

A number of agents that selectively induce cytotoxicity and eliminate diseased cells are currently under development. These agents range from small molecules or biologics to cytolytic immune effector cells genetically engineered to selectively recognize tumor associated antigens (TAA). Successful selection and optimization of these agents depends on the accuracy and sensitivity of assays employed to measure cytotoxicity.

Several assays have been developed to measure cytotoxicity. Of these, radio-active chromium (Cr^51^) release assay developed in 1968 is most commonly used worldwide^1^. In this assay, target cells labeled with Cr^51^ are incubated with effector cells and Cr^51^ released upon their lysis serves as a measure of the effector cell cytotoxicity. However, several limitations including the hazards associated with harmful effects of radioactivity, additional costs of disposal of radioactive waste and requirement of additional equipment like gamma counters, have prompted researchers to seek safer alternative approaches. For example, cell membranes of target cells can be labeled with fluorescent dyes and cytotoxic response can be evaluated using multicolor flow cytometric analysis^2^. However, the successful application of this approach demands careful calibration and labor intensive data analysis to efficiently distinguish the target and effector cell populations.

Living cells exclude vital dyes such as trypan blue. Loss of cell membrane integrity not only allows the vital dyes to enter the cell but also results in release of cytoplasmic components into the surrounding medium. Some cytotoxicity assays are based on quantification of the release of cytosolic enzymes such as lactose dehydrogenase (LDH)^3^, glyceraldehyde 3-phosphate dehydrogenase (G3PDH)^4^ or adenylate kinase (AK)^5^ from dead cells. All these assays measure enzyme activity either directly by providing substrates that would be converted to fluorescent or luminescent products or include a second step wherein products of the primary reaction indirectly generate substrate for a luciferase reaction. Most of these enzymatic methods require a two-step procedure to remove culture medium to a separate container and thus are non-homogeneous. Additionally, these methods, in general, have poor sensitivity and, importantly, are unable to distinguish between death of target and effector cells, since both types of cells release cellular enzymes upon lysis.

Luciferases have been used extensively as reporters because of their ability to provide highly sensitive quantitation with broad linearity^6^. Firefly (Fluc) and Renilla (Rluc) luciferases have accounted for the majority of such applications^7^. A luciferase release-based cytotoxicity assay was first described by Schafer *et al*. using Fluc^8^. However, the shorter half-life of Fluc (<30 minutes) in tissue culture medium hindered its wider use^8,9^. Fu *et al*.^10^ also tried to develop a T cell cytotoxicity assay based on measurement of Fluc that has been released in the medium of Fluc-expressing target cells upon incubation with cytotoxic T cells. Surprisingly, incubation of cytotoxic T cells with the tumor cell targets did not result in significant release of luciferase in the culture medium^10^. Therefore, the idea of measuring release of luciferase in the supernatant as an assay for cytotoxicity was abandoned^8,10^.

A number of novel luciferases have been discovered from deep sea marine organisms. These marine luciferases are smaller in size, ATP-independent and have much brighter luminescence and stability as compared to Fluc^11^. All of them have an N-terminal 17–22 amino acid consensus sequence that signals secretion^11^.

In this study, we describe the development of a novel cytotoxicity assay, termed Matador assay, which is based on increase of reporter activity of cytosolic sequestered variants of reporters, such as marine luciferases, from dead and dying cells. The Matador assay is based on the fact that loss of cell membrane integrity results not only in the release of cytosolic sequestered reporters into the surrounding medium but also in the greater and faster penetration of the reporter substrate into the cell where it can react with any reporter still trapped inside the cell. The Matador assay takes advantage of the longer half-life (>24 hours), stability and extreme brightness of the marine luciferases and their engineered derivatives. The assay is non-radioactive, extremely sensitive, inexpensive, homogeneous and amenable to miniaturization and automation.

## Results

### Development of a cytotoxicity assay based on transient expression of different luciferases in cytosol

Gaussia luciferase (Gluc; 185 aa, 19.9 kDa) is one of the smallest luciferase known and is naturally secreted^12^. We hypothesized that deletion of signal sequence of Gluc will result in the entrapment of the protein inside the cells. Treatment of cells with intracellular expression of Gluc with cytotoxic cells or agents will compromise cell membrane integrity, resulting in the release of entrapped Gluc, which would serve as a readout for cell death. To test our hypothesis, we cloned the cDNA of Gluc lacking its signal peptide (SP) into a retroviral vector (MSCV-hygro-Gluc). We also expressed the Gluc cDNA lacking the signal peptide from lentiviral vectors pLenti (expression driven by CMV promoter) and pLenti-EF1α (expression driven by the constitutively active Elongation Factor 1-alpha promoter). The construct pLenti-Pac-Gluc, also expressed the puromycin resistance gene (Pac) upstream of and in-frame with the Gluc cDNA and separated from the latter by a T2A ribosomal skip sequence.

**Figure 1:**
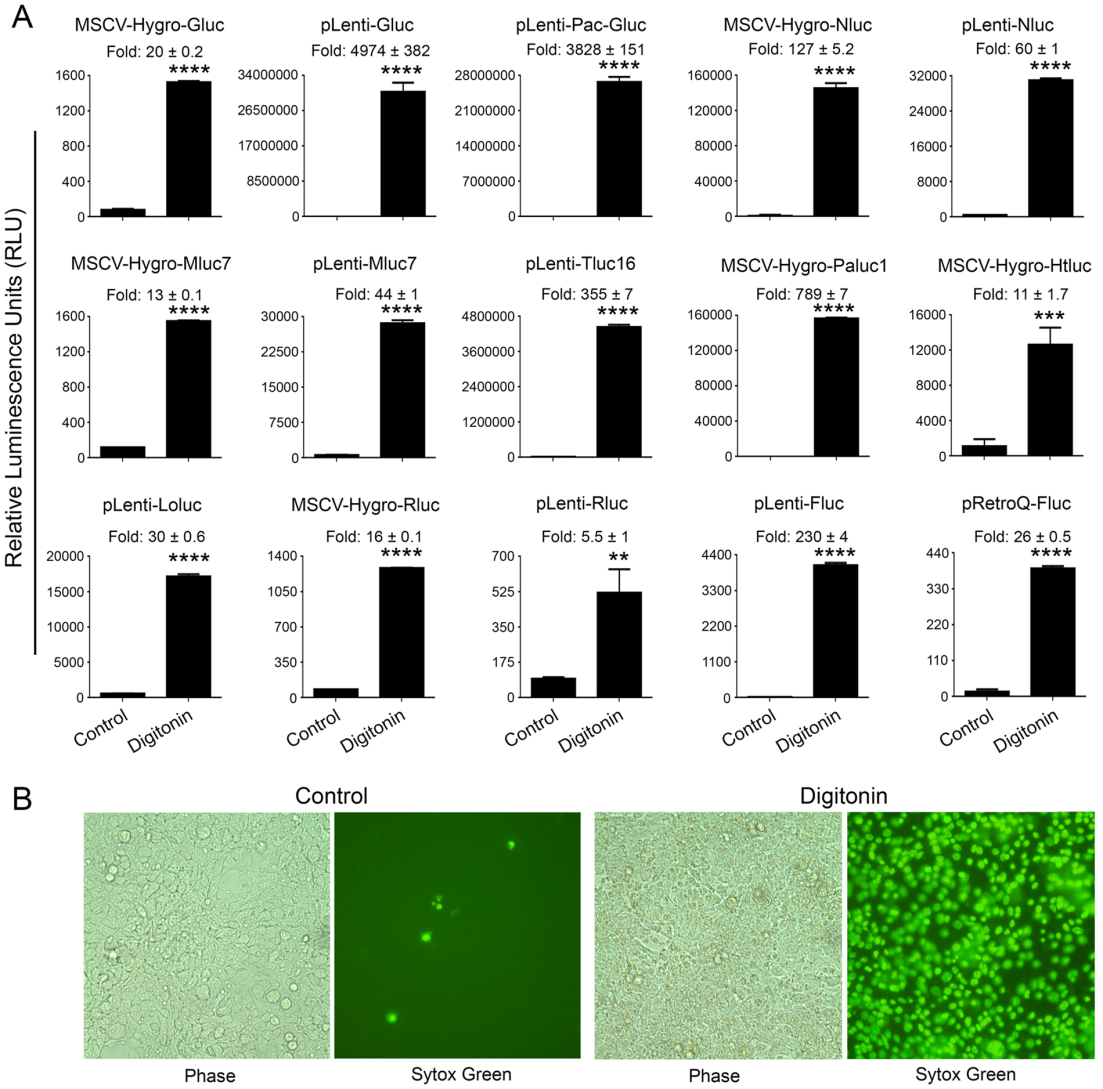
Performance of the Matador assay in transient transfection. (**A**) The indicated luciferase constructs in either retroviral or lentiviral vectors were transiently transfected in 293FT cells. Approximately, 18 hours post-transfection cells were treated with digitonin (30 µg/ml) for 90 minutes or left untreated (control). Cell-free supernatants (25 µl) were assayed for luminescence by addition of coelenterazine containing assay buffer (25 µl) directly to each well in a 384-well lumitrac plate for Gluc, Nluc, Mluc7, Tluc16, Paluc1, Htluc1, Loluc and Rluc. For Fluc, D-Luciferin containing assay buffer was used. The fold change in the relative luminescence units is given on top of each graph. The values shown are mean ± SE of a representative experiment performed in duplicate for at least two times. Statistically significant differences are shown by asterisks (**) at a level of *P* < 0.01 and (****) at a level of *P* < 0.0001. (**B**) Confluent layer of 293FT cells were treated with digitonin (30 µg/ml) for 90 minutes, followed by the addition 1 μM sytox green, a cell-impermeable nuclear dye that stains the nuclei of dead cells were examined under a fluorescence microscope or under phase-contrast microscope and photographed.

We transiently transfected the above vectors into 293FT cells. Approximately 24 hours post-transfection, cells were treated with 30 µg/ml of digitonin for 90 minutes to induce cell death. Treatment of 293FT cells transfected with SP-deleted Gluc with digitonin resulted in a dramatic increase of Gluc activity in the supernatant (Fig. 1A). In particular, digitonin-treatment of cells transfected with MSCV-Hygro-Gluc, pLenti-Gluc, and pLenti-Pac-Gluc resulted in 20-, 4974-, and 3828-fold increase in Gluc activity, respectively, as compared to the untreated cells. To examine whether this phenomenon is extendable to other luciferases, we cloned SP-deleted versions of Nluc, Tluc16, Mluc7, Paluc1, Loluc and Htluc1 into lenti- and/or retro-viral vectors. The constructs were transfected into 293FT cells and treated with digitonin to induce cell death. Consistent with the previous results, treatment with digitonin resulted in 127-, 60-, 13-, 44-, 355-, 789-, 11-, and 30-fold increase in luciferase activity in the supernatant of cells transfected with MSCV-Hygro-Nluc, pLenti-Nluc, MSCV-Hygro-Mluc7, pLenti-Mluc7, pLenti-Tluc16, MSCV-Hygro-Paluc1, MSCV-Hygro-Htluc1 and pLenti-Loluc, respectively (Fig. 1A). Interestingly, treatment with digitonin also resulted in a significant increase in luciferase activity in the supernatant of cells transfected with expression vectors encoding commonly used renilla (Rluc) and firefly (Fluc) luciferases with fold increases of 16, 5.5, 26, and 230 for MSCV-Hygro-Rluc, pLenti-Rluc, pRetroQ-Fluc, and pLenti-Fluc, respectively (Fig. 1A, bottom panel). Cell death induction by digitonin was further confirmed by increased staining with sytox green, a cell impermeable dye that stains cells that have lost membrane integrity (Fig. 1B). Collectively, the above results suggest that potentially all luciferases can serve as candidates for the development of Matador cytotoxicity assay.

### Performance of cytotoxicity assay on cancer cell lines stably expressing various luciferases

**Figure 2:**
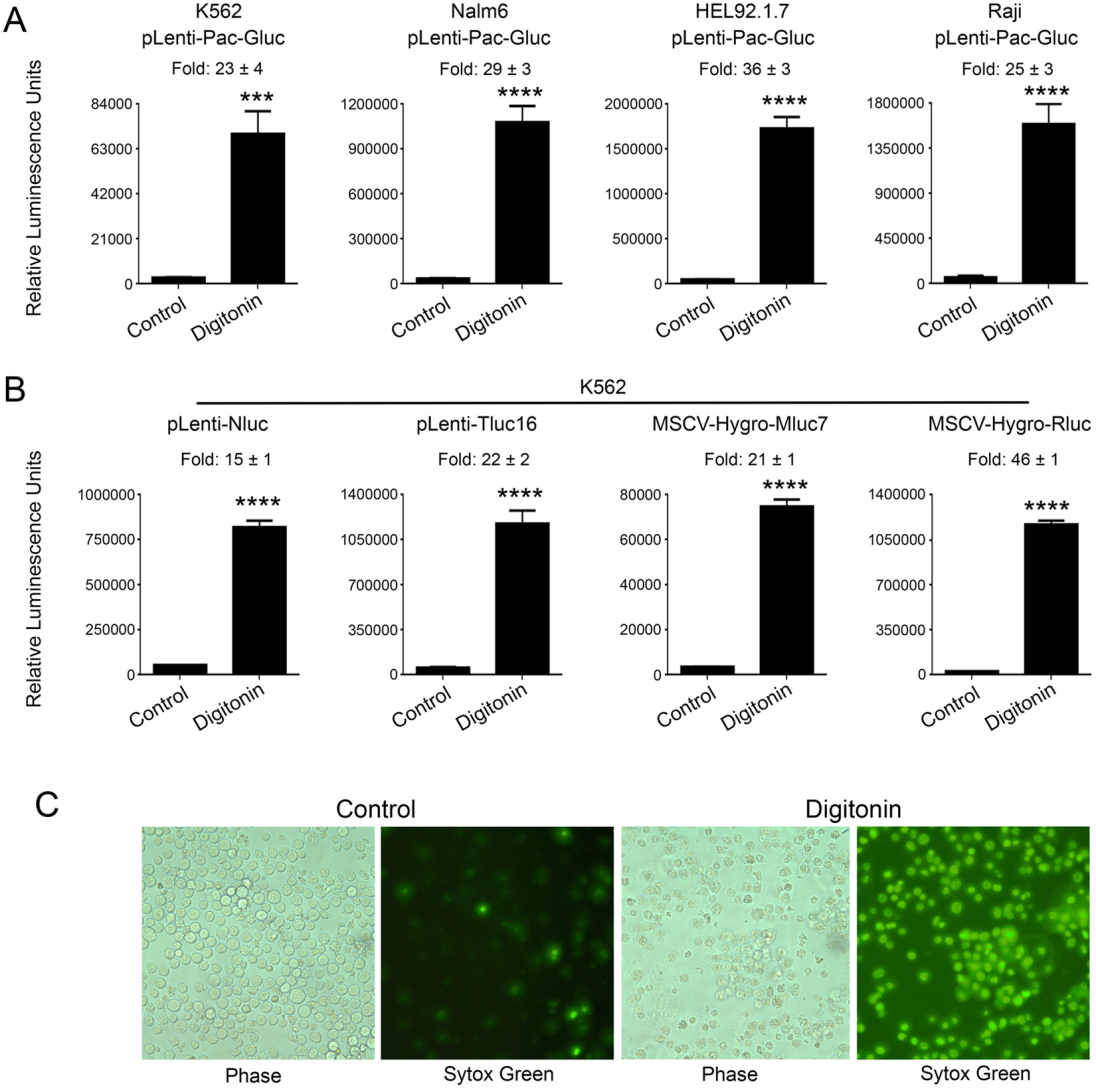
Performance of the Matador assay in cancer cell lines stably transduced with various luciferases. (**A**) Indicated cell lines stably transduced with pLenti-Pac-Gluc virus were plated in a 384 well Lumitrac plate and treated with digitonin (30 µg/ml) for 90 minutes or left untreated (control). Luminescence was detected by the addition of coelenterazine containing assay buffer directly to each well. Statistically significant differences are shown by asterisks (***) at a level of *P* < 0.001 and (****) at a level of *P* < 0.0001. (**B**) K562 cells stably expressing Nluc/Tluc16/Mluc7/Rluc were treated with digitonin and luminescence was detected as described earlier. The values shown are mean ± SE of a representative experiment performed in triplicate for at least two times. (**C**) K562 cells were treated with digitonin (30 µg/ml) for 90 minutes, followed by the addition 1 μM sytox green. Cells were examined under a fluorescence microscope or under a phase-contrast microscope and photographed.

We next tested the feasibility of our assay in cancer cell lines stably expressing various luciferases. For this purpose, we infected K562, Nalm6, HEL92.1.7 and Raji cells with pLenti-Pac-Gluc virus and selected stably transduced cells with puromycin. Treatment of Gluc-expressing K562, Nalm6, HEL92.1.7 and Raji cells with digitonin resulted in 23-, 29-, 36- and 25-fold increase of Gluc activity, respectively, as compared to the untreated cells (Fig. 2A). Essentially similar results were obtained with K562 cells stably transduced with pLenti-Nluc, plenti-Tluc16, MSCV-Hygro-Mluc7, and MSCV-Hygro-Rluc (Fig. 2B). The death of K562 cells after treatment with digitonin was confirmed by sytox-green staining (Fig. 2C). These results demonstrate that the Matador assay can be performed on cell lines stably expressing the different luciferases.

### Sensitivity of the Matador assay

**Figure 3:**
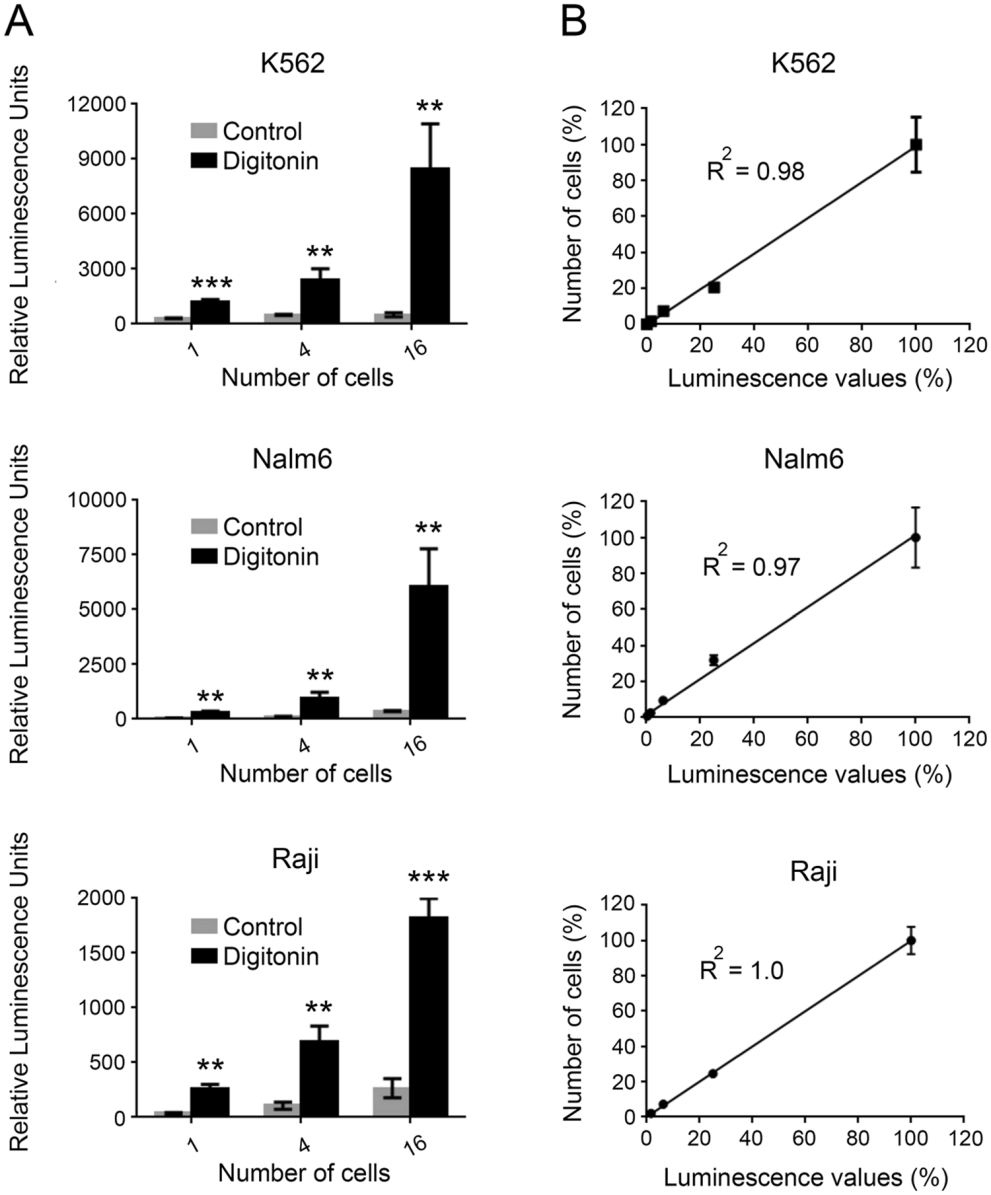
Sensitivity of the Matador assay. (**A**) Indicated cell lines stably expressing pLenti-Pac-Gluc were placed in a 384-well plate at indicated numbers (by serial dilution), and treated with digitonin (30 µg/ml) for 120 minutes or vehicle (control). Luminescence was detected by the addition of coelentrazine containing assay buffer directly to each well. Statistically significant differences are shown by asterisks (**) at a level of *P* < 0.01 and (***) at a level of *P* < 0.001. Statistical differences shown are between control *vs* digitonin treated samples. (**B**) Linear increase in luminescence over a wide range of cell numbers in the Matador assay. Both the number of cells plated and luminescence values detected were converted into percentage by dividing the individual values with the maximum cell numbers plated (4096) or the luminescence values from the well with maximum number of cells, respectively. R^2^ = Correlation coefficient. The values shown are mean ± SE of a representative experiment performed in triplicate for at least two times.

To determine the sensitivity of the Matador assay, we plated different cell concentrations of K562, Nalm6 and Raji cells stably expressing Gluc followed by treatment with digitonin to induce cell death. Strikingly, in all the tested cell lines, there was a significant increase in the luciferase activity upon induction of cell death (Fig. 3A). Importantly, the increase in luciferase activity was evident even in wells that contained a single cell (Fig. 3A). Moreover, there was a near linear increase in the luciferase values with increase in cell numbers (R^2^ ≥ 0.97) (Fig. 3B). Essentially, similar results were obtained with Raji, K562, and HEL92.1.7 cells stably expressing Nluc, or Mluc7 (Supplementary Fig. S1), highlighting the extreme sensitivity and broad applicability of the assay.

We also compared the sensitivity of the Matador assay with LDH and Calcein-release assays, two cytotoxicity assays that are in common use. In contrast to single cell sensitivity of the Matador assay, the minimum number of cells that could be detected with the LDH and the Calcein-release assays were 256 and 64, respectively (Supplementary Figs S2 and S3). Thus, the Matador assay possesses greater sensitivity as compared to the LDH- and Calcein-release assays.

### The Matador assay is a single step homogenous assay

**Figure 4:**
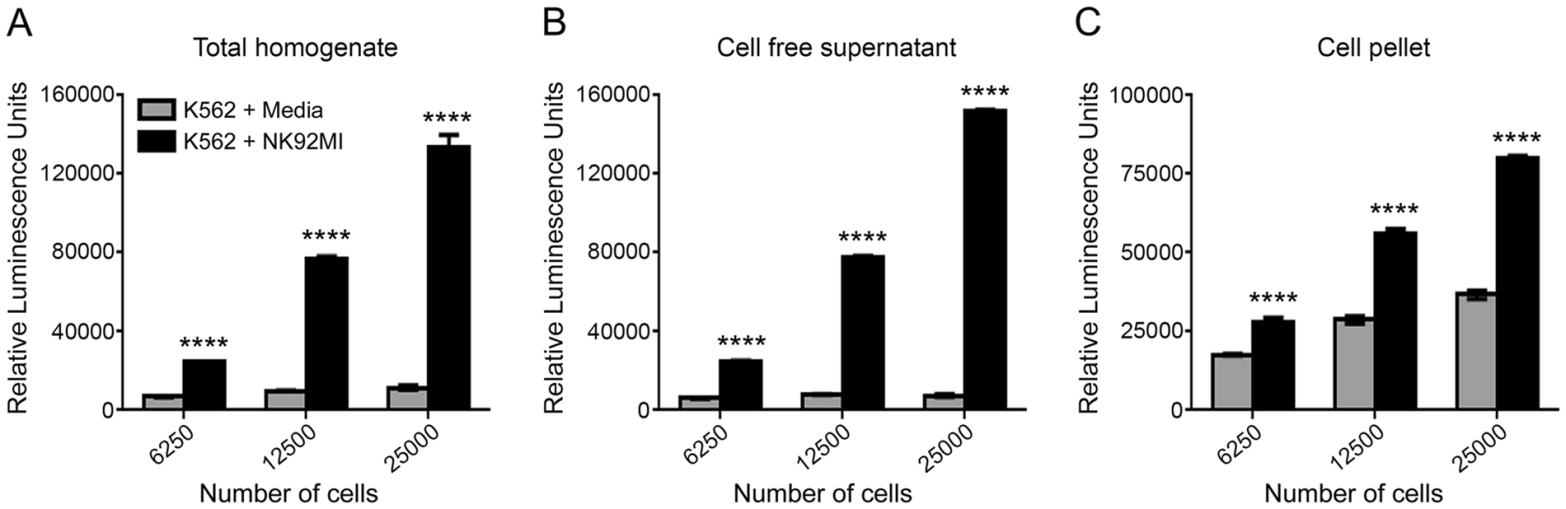
The Matador assay is a single step homogenous assay. K562 cells stably expressing Gluc were co-cultured with NK92MI in a 24-well plate at an Effector:Target (E:T) ratio of 0.5:1 for 4 hours. Post-incubation, the cells were mixed well, collected in a 1.5 ml microfuge tubes and divided into 3 parts. (**A**) (Total/homogeneous), the cells along with supernatant were directly assayed for luciferase activity by plating in a 384-well plate. (**B**) (Cell free supernatant), cells were centrifuged and cell supernatants alone were collected in a new tube and plated in a 384-well plate, followed by measurement of the luciferase activity. (**C**) (Cells Pellet), cells were centrifuged, supernatant was removed, cell pellets were resuspended in PBS and plated in a 384-well plate to measure luciferase activity. Total reaction volume plated in 384-well plate for all 3 parts was kept constant at 50 µl per well and 15 µl of coelenterazine assay buffer was added in well mode to measure luciferase activity. The three figures are from a single experiment and therefore the values can be compared across the three figures for comparison. The values shown are mean ± SE of a representative experiment performed in triplicate. Statistically significant difference is shown by asterisks (****) at a level of *P* < 0.0001. Statistical differences shown are between K562 + Media *vs* K562 + NK92MI.

A single-step homogenous assay, which does not involve a centrifugation step to separate the cells from supernatant, has obvious advantages for miniaturization and automation. Most of the experiments described in the preceding sections (Figs 2 and 3) were done in a homogeneous manner. To assess whether separation of supernatants from cell pellets alters the sensitivity of the assay, we performed the assay in cell-free supernatant, cells alone and in total homogeneous mixture. For this purpose, K562 cells stably expressing Gluc were co-cultured with the natural killer-derived cell line NK92MI at an Effector:Target (E:T) ratio of 0.5:1 for 4 hours, followed by the measurement of Gluc activity in the different fractions. Figure 4(A,B) shows that induction of K562-Gluc cell death by NK92MI cells resulted in significant and near equivalent increases in Gluc activity when measured directly by adding coelenterazine (CTZ) assay buffer to the wells containing the cells or to the wells containing cell-free supernatant. These results demonstrate that the centrifugation step to collect the cleared supernatant can be omitted without compromising the sensitivity of the assay, thereby yielding a single-step homogenous assay. We also observed a slight increase in Gluc activity when the assay was performed on the cell pellet of K562-Gluc cells that had been co-cultured with NK92MI cells (Fig. 4C). This could reflect greater penetration of CTZ in cells with impaired cell membrane integrity.

### Marine luciferase and their engineered derivatives are highly stable in cell culture supernatants and are ideal for the Matador assay

**Figure 5:**
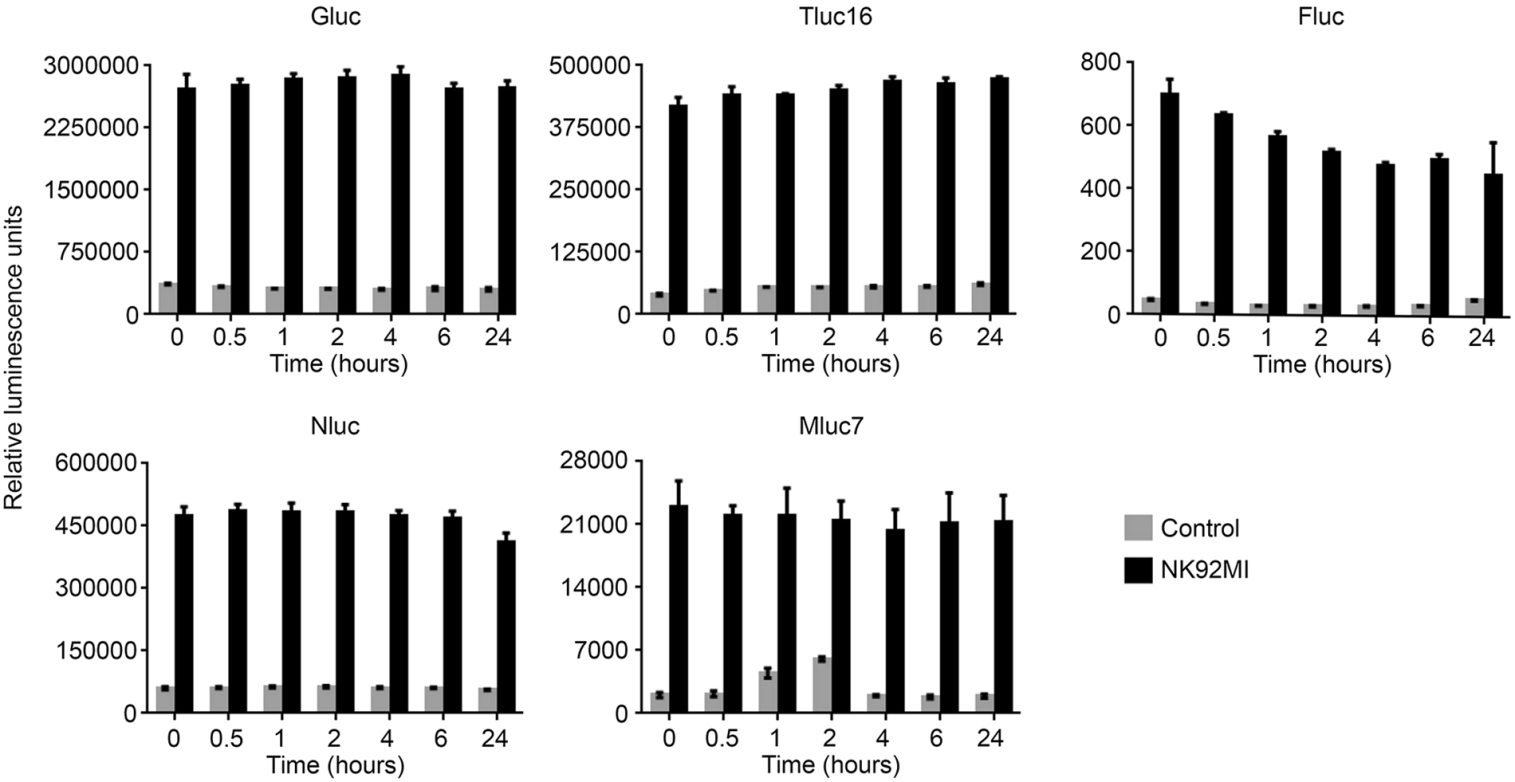
Stability of different luciferases used in the Matador assay. K562 cells stably expressing Gluc/Fluc, Nluc, Tluc16, and Mluc7 were plated in a 24-well plate, treated with media alone (control) or co-cultured with NK92MI at Effector:Target (E:T) ratio of 1:1 for 6 hours. After incubation, cell free supernatants were transferred into 7 different tubes and frozen immediately at −80 °C. The tubes were directly transferred to 37 °C (from −80 °C) and were incubated for indicated time periods (0–24 hours). The luminescence was measured by adding coelenterazine-containing assay buffer (for Gluc, Nluc, Tluc16, and Mluc7) and D-luciferin containing assay buffer (for Fluc) directly to each well. The values shown are mean ± SE of a representative experiment performed in duplicate for at least three times.

Although the development of a cytotoxicity assay based on the release of Fluc in the cellular supernatant has been tried in the past, the short half-life of Fluc in the tissue culture medium precluded its successful development^8^. The marine luciferases used in this study have been reported to have longer half-life (>24 hours) in tissue culture medium (Table 1). However, we wanted to confirm that they would retain their stability under the assay conditions and will not get degraded by the proteases released during the process of cell death. Therefore, to demonstrate that the marine luciferases are suitable for the Matador assay, K562 cells stably expressing Gluc, Nluc, Tluc16, Mluc7 and Fluc, were plated in a 24-well plate and treated with media alone or with NK92MI cells at an Effector:Target (E:T) ratio of 1:1 for 6 hours. Post-incubation, the cell-free supernatants were divided into 7 different tubes and frozen immediately at −80 °C. The tubes were then incubated at 37 °C for 0, 0.5, 1, 2, 4, 6, and 24 hours. After incubation, supernatants were transferred to a 384-well plate, and luminescence was measured by adding CTZ-containing assay buffer (for Gluc, Nluc, Tluc16, and Mluc7), and D-luciferin-containing assay buffer (for Fluc). As shown in Fig. 5, luminescence of Gluc, Nluc, Tluc16, and Mluc7 was stable for 24 hours at 37 °C in cell culture supernatants, demonstrating the suitability of these luciferases for the Matador assay. In contrast, luminescence of Fluc started decaying as early as 0.5 hours, confirming that Fluc may not be a suitable candidate for the Matador assay.

**Table 1:** List of luciferases tested in this study and their general characteristics.

Luciferase	Species	Substrate	Type of luminescence	MW (kDa)	ATP-Dependent	Secretory	Half-life (cell culture conditions)
Gluc^12,25^	*Gaussia princeps*	CTZ	Flash	20	No	Yes	>6 days^12,25^
Nluc^7^	*Oplophorus gracilorostris*	CTZ	Flash and engineered for glow	19	No	Yes	>7 days^7^
Tluc16	*Metridia* family	CTZ	Flash and engineered for glow	16	No	Yes	>24 hours ^(This study)^
Mluc7^19^	*Metridia longa*	CTZ	Flash	18	No	Yes	>24 hours^19^
Loluc^11^	*Lucicutia Ovaliformis*	CTZ	Flash	24	No	Yes	ND
Paluc1^11^	*Plueromamma abdominalis*	CTZ	Flash	23	No	Yes	ND
Htluc1^11^	*Heterorhabdus tanneri*	CTZ	Flash	21	No	Yes	ND
Rluc	*Renilla reniformis*	CTZ	Flash	36	No	No	57 minutes^32^
Fluc	*Photinus pyralis*	D-Luciferin	Glow	61	Yes	No	30 minutes^8,9^

Abbreviations: CTZ – Coelenterazine; MW – Molecular weight; kDa – Kilo Daltons; ND – Not Detected.

### Ectopic expression of marine luciferases does not alter the sensitivity of the transduced cells to cytotoxic agents

**Figure 6:**
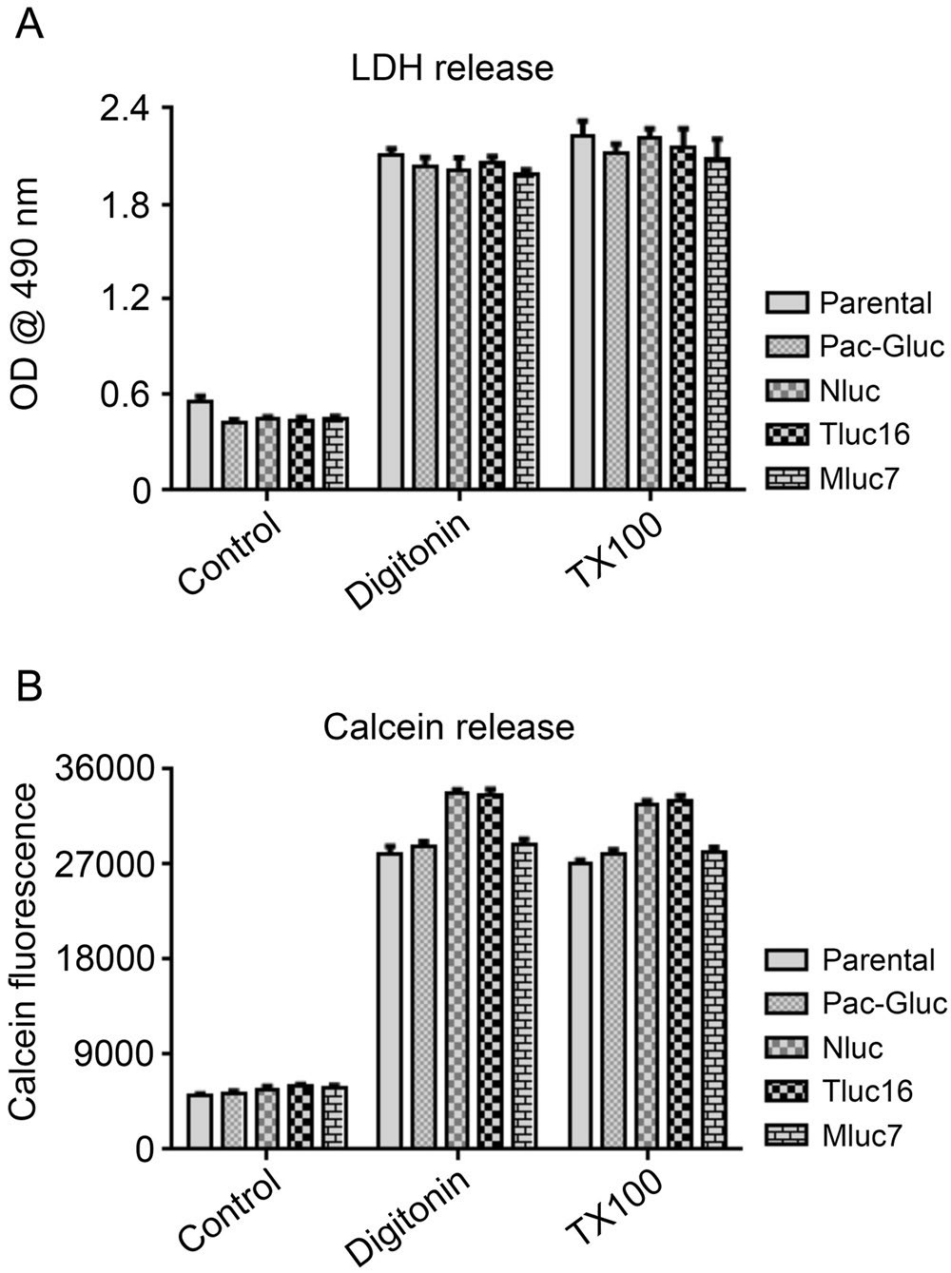
Expression of marine luciferases does not alter target cells sensitivity to cytotoxic agents. (**A**) LDH release assay. K562 cells stably expressing Gluc, Nluc, Tluc16, Mluc7, and parental line were plated in a 96-well U bottom plate in phenol red free media, followed by treatment with media alone (control), digitonin (30 µg/ml for 90 minutes), or 1% Triton X-100 (TX100) for 45 minutes. Post incubation the plates were spun at 250 *g* for 4 minutes. Supernatants were carefully transferred into a flat bottom plate followed by the calorimetric detection of LDH activity as described in the manufacturer protocol. (**B**) Calcein release assay. Indicated cell lines were pre-incubated with Calcein-AM as described in methods section and the experiment set up was same as for LDH release assay, except that the supernatants were transferred into a black walled flat bottom plate followed by measurement of fluorescence (Excitation 485 ± 9 nm, Emission 530 ± 9 nm) in a Biotek Synergy plate reader.

It is conceivable that expression of marine luciferases may alter the sensitivity of target cells to cell death inducing agents. In order to rule out this possibility, K562 cells stably expressing Gluc, Nluc, Tluc16, Mluc7, and parental line were treated with digitonin and Triton X-100. The cell death was measured by LDH and Calcein-release assays. As shown in Fig. 6A,B, the parental and the marine luciferases-transduced K562 cells have similar sensitivity to cytotoxicity induced by digitonin and Triton X-100, as measured by two assays that are independent of the measurement of luciferase activity. These results demonstrate that marine luciferase gene transduction did not alter the sensitivity of cells to cytotoxic agents. Further, we have made more than 80 cell lines stably transduced with marine luciferases and have not observed any significant changes in their morphology, growth rate, sensitivity to chimeric antigen receptors expressing T cells or other characteristics in comparison to their parental cell lines.

### Exemplary applications of the Matador assay in the areas of cellular- and immuno-therapy

**Figure 7:**
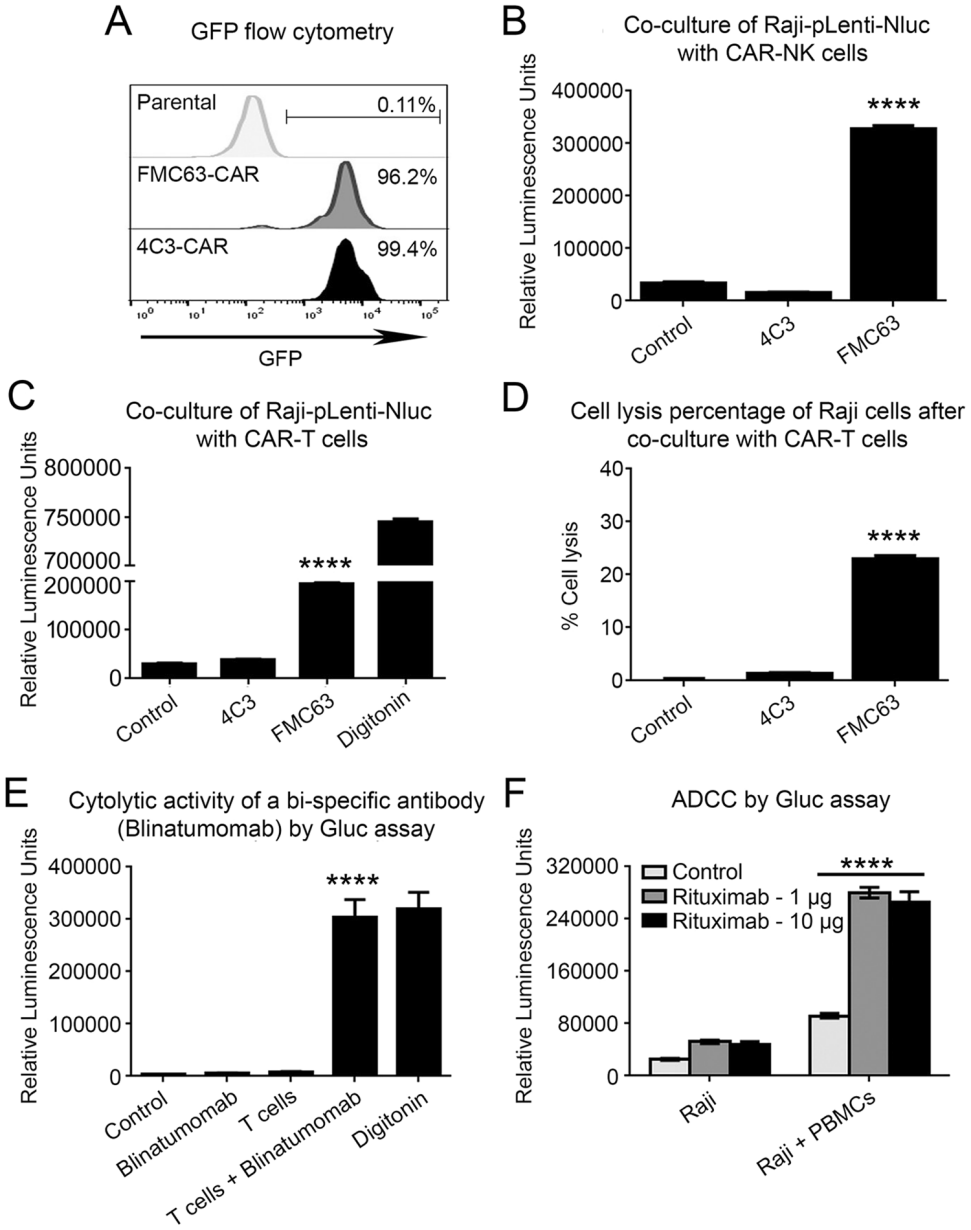
Applications of the Matador assay for measuring cytotoxicity induced by CAR-T cells, Blinatumomab and Rituximab. (**A**) Flow cytometry analysis of NK92MI cells stably transduced with chimeric antigen receptors targeting CD19 (FMC63) or a control CAR based on detection of co-expressed eGFP expression. (**B**) Raji cells stably expressing Nluc were co-cultured in a 384-well plate at an E:T ratio of 0.5:1 for 4 hours with NK92MI cells expressing the indicated CARs in 60 μl total volume. Post-incubation, luminescence was detected by the addition of 15 µl of coelenterazine-containing assay buffer directly to each well. (**C**) Primary human T-cells transduced with the indicated CARs were co-cultured in a 384-well plate at an E:T ratio of 10:1 for 4 hours with Raji cells stably expressing Nluc, followed by the detection of luminescence as described earlier. (**D**) Bar graph depicting the percentage specific lysis of Raji cells by indicated CAR-Ts. The luminescence values of cells incubated with media alone is considered as 0% (spontaneous cell death or basal) and the digitonin-induced cell death is considered as 100% (maximum cell death or total). (**E**) Raji cells stably expressing Gluc were treated with Blinatumomab at a concentration of 100 ng/10^6^ cells for 30 minutes at 37 °C in PBS containing 2% FBS. Next, antibody-treated cells were washed three times in PBS-FBS, re-suspended in XVIVO-15 medium and co-incubated with primary human T cells at a E:T ratio of 20:1 for 4 hours. Luminescence was detected by the addition of coelenterazine-containing assay buffer directly to each well. (**F**) Raji cells stably expressing Gluc were co-cultured with peripheral blood mono nuclear cells (PBMCs) from healthy donors at an E:T ratio of 40:1 for 4 hours in the presence of indicated concentrations of Rituximab, followed by the detection of luminescence as described earlier. The values shown are mean ± SE of a representative experiment performed in triplicate for at least two times. Statistically significant difference is shown by asterisks (****) at a level of *P* < 0.0001.

Cytotoxic assays are frequently used in the fields of cellular- and immune-therapy, such as to test the cytotoxicity of chimeric antigen receptor (CAR)-expressing T cells, Bispecific T cell Engagers (BiTE) and cytotoxic antibodies. However, a problem with most currently used cytotoxic assays, such as the LDH release assay, is their inability to distinguish between the death of the target cells from death of the effector cells. To demonstrate the utility of the Matador assay in co-culture experiments, we generated NK92MI cells stably expressing a chimeric antigen receptor (CAR), designated FMC63-CAR, targeting CD19. We also generated a CAR targeting a protein encoded by the Kaposi’s sarcoma herpesvirus (KSHV), designated 4C3-CAR, to serve as a control. The expression vectors encoding the CARs also co-expressed Green Fluorescent Protein (GFP), which was used to confirm expression of the CAR construct in NK92MI cells using flow cytometry analysis (Fig. 7A). As shown in Fig. 7B, co-culture of CD19^+ve^ Raji cells stably expressing Nluc (Raji-pLenti-Nluc) with FMC63-CAR-expressing NK92MI cells resulted in a robust increase in the Nluc activity, whereas co-culture with the control 4C3-CAR-expressing NK92MI cells failed to do so (Fig. 7B). Essentially similar results were obtained upon co-culture of Raji-pLenti-Nluc cells with primary T cells that had been transduced with the different CAR constructs (Fig. 7C). We also calculated the percentage-specific lysis induced by different CAR constructs (Fig. 7D) by taking the Nluc activity of digitonin-treated cells as a measure of maximum cell death and Nluc activity of untreated cells as a measure of spontaneous cell death. The percent specific lysis of Raji-pLenti-Nluc cells induced by FMC63-CAR-T cells was approximately 20%.

We next tested the ability of the Matador assay to detect Blinatumomab- and Rituximab- induced cytotoxicity. Blinatumomab is a bispecific T cell engager (BiTE) which has binding sites for both CD19 (present on B-lineage lymphoid cells) and CD3 (present on T-cells), and results in the activation of T-cells and destruction of CD19^+^ cells^13^. Co-culture of Raji-Gluc cells with T-cells in the presence of Blinatumomab resulted in a striking increase in Gluc activity as compared with Raji-Gluc cells treated with Blinatumomab alone or untreated T-cells (Fig. 7E). Rituximab is a CD20 monoclonal antibody that is in clinical use for a number of B cell malignancies^14^. Rituximab induces antibody-dependent cell mediated cytotoxicity of CD20^+^ cells. As shown in Fig. 7F, co-culture of Raji-Gluc cells with peripheral blood mononuclear cells (PBMCs) in the presence of Rituximab resulted in a robust increase in Gluc activity as compared to cells in which either PBMCs or Rituximab was excluded. Taken collectively, the above results demonstrate the utility of the Matador assay to detect cytotoxicity induced by a number of cellular and biological therapeutics.

### Primary cells and cell lines transiently expressing luciferase reporters can be used in the Matador assay

**Figure 8:**
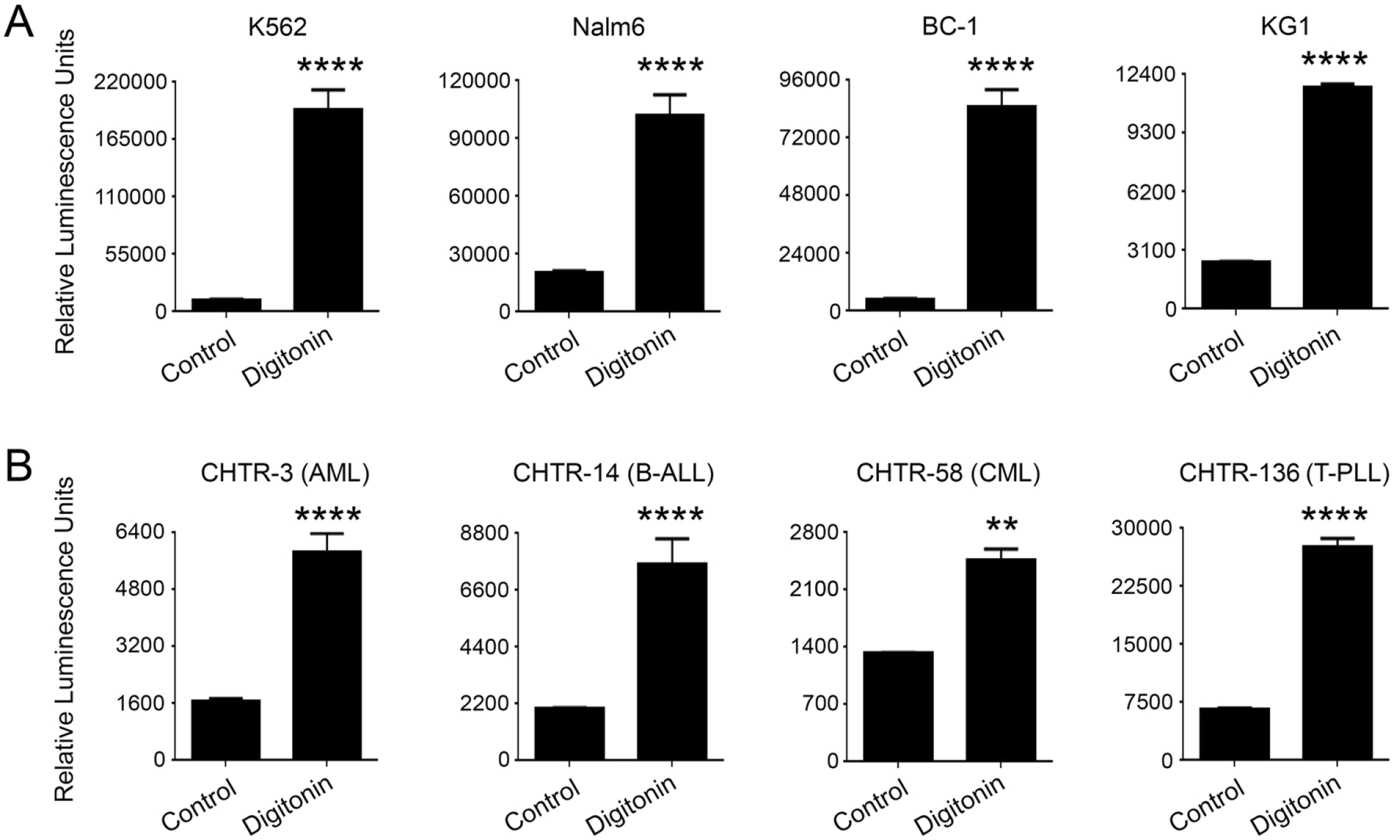
Feasibility of Matador assay in primary cells and cell lines transiently expressing a luciferase reporter. (**A**) Indicated cell lines were transduced with pLenti-Pac-Gluc (MOI-10) 48 hours post-transduction, cells were plated in a 384 well plate and treated with digitonin (30 µg/ml) for 120 minutes or media alone (control). Luminescence was detected by the addition of coelenterazine containing assay buffer directly to each well. (**B**) Leukemic cells were transduced with pLenti-Pac-Gluc (MOI-10). and Matador assay was performed in a similar manner as described for A. Statistically significant differences are shown by asterisks (**) at a level of *P* < 0.01 and (***) at a level of *P* < 0.001. Abbreviations: AML, Acute Myelogenous Leukemia; B-ALL, B cell Acute Lymphoblastic Leukemia; CML, Chronic Myelogenous Leukemia; T-PLL, T cell Pro-lymphocytic Leukemia. CHTR, Comprehensive Hematology Tissue Repository.

In the preceding examples, we demonstrated the feasibility of performing the Matador assay on cell lines that were stably transduced with the various marine luciferases. Since the generation of stably transduced cell lines takes several weeks, we tested the feasibility of performing the Matador assay on cell lines that had been transduced with marine luciferase expressing viral vectors but had not undergone the time-consuming step of drug selection to select stably transduced clones. K562, Nalm6, BC-1, and KG-1 cells were transiently transduced with the pLenti-Pac-Gluc viral vector and 48 hours post-transduction treated with digitonin to induce cell death. Digitonin-treatment resulted in striking increases in the luminescence signal of all the cell lines that were transduced with pLenti-Pac-Gluc (Fig. 8A). Essentially similar results were obtained when Raji and K562 cells were transiently transfected with a Gluc encoding plasmid and cell death was induced by treatment with digitonin, FMC63-CAR-T or NK92MI cells (Supplementary Fig. S4).

Finally, we tested the feasibility of Matador assay on primary cells. Primary leukemia cells isolated from patients with Acute Myeloid Leukemia (AML), B-cell lymphocytic leukemia (B-ALL), chronic myeloid leukemia and T cell prolymphocytic leukemia (T-PLL) were transduced with pLenti-Pac-Gluc lentivirus supernatant. Approximately 48 hours post-transduction, cell death was induced by treatment with digitonin. A significant increase in the luminescence signal was observed in all the Gluc-expressing primary samples upon the induction of cell death (Fig. 8B).

## Discussion

Recent success in clinical trials using chimeric antigen receptor (CAR) targeting CD19 for B cell malignancies and the FDA approval of Blinatumomab have revived hopes for immunotherapeutic approaches to treat cancer^15–17^. Numerous academic laboratories and pharmaceutical companies are now developing CARs and BiTEs with predefined specificity to desired tumor associated antigens (TAA) for adoptive cellular therapy. The success of these efforts and prior conventional approaches using tumor specific T-cell receptors largely relies on availability of a simple, accurate and robust assay to rapidly assess the ability of the immune effector cells to kill tumor cells expressing desired TAA.

The Cr^51^ assay has been used as the “gold standard” for evaluating cell mediated cytotoxicity for nearly five decades^1^. This assay demands prior loading of target cells with radioactive Cr^51^, which is released into the culture medium upon cell lysis mediated by the effector cells. In addition to health risks associated with radioactivity, need for specialized training, expensive equipment and the burden of safe disposal of radioactive waste, this method is technically laborious. Labelling of cellular proteins by Cr^51^ is non-specific and complete release of bound Cr^51^ from lysed cells is inefficient, leading to low signal-to-noise ratio^18^. To circumvent these issues associated with exogenous chemical agents, other assays that monitor release of endogenous enzymes, such as LDH and G3PDH, were developed^3–5^. However, as these enzymes are endogenously expressed in both effector and target cells, measurement of their release as a measure of cellular toxicity can be inaccurate in a heterogeneous mixture. In particular, activation induced cell death of effector cells, which are present in excess in cytotoxicity assays, can confound the interpretation of results. Though alternative approaches using fluorescent dyes have been developed^2^, difficulties associated with efficient preloading and spontaneous release from target cells still pose technical challenges.

To overcome the above limitations, an attempt was made to develop a luciferase-release based cytotoxicity assay using Fluc^8^. However, it was observed that the half- life of Fluc under culture conditions is only about 30 minutes so that after 4 hours almost 80% of the original enzyme is inactive and no longer detectable^8^. This precluded the use of Fluc as a reporter for cytotoxicity assays requiring more than 2 hours of incubation. Furthermore, it was observed that it is not informative to calculate the released Fluc enzyme activity relative to the 100% lysis value because depending on the kinetics of the release, variable proportions of the released enzyme will be inactive at the time of removal of the supernatant for measurement^8^. An additional limitation of Fluc-release based cytotoxicity assay was discovered in a study by Fu *et al*.^10^. These investigators were not able to detect luciferase activity in the cell culture supernatant of 4T1-Her2 cells that stably-expressed Fluc upon T cell mediated cytolysis. Due to the above limitations of short half-life and inconsistency of detection, Fluc-based reporter release assay is not routinely used for measuring cell-mediated cytotoxicity.

The Matador assay described in this study uses marine luciferases and has several distinct advantages over Fluc-based reporter release assays described previously. First, the assay takes advantage of the extreme brightness and ultra-sensitivity of the marine luciferases, which is, in general, several orders of magnitude higher than Fluc^7,12^, making it an extremely sensitive assay, In fact, the Matador assay is capable of detecting cell death at a single cell level. Second, in contrast to the short half-life of Fluc, marine luciferases used in this study are highly stable in cell culture media (t_1/2_ > 24 hours). Therefore, the Matador assay is ideal for most cytotoxicity assays which are generally carried over durations of 4 hours or more. Third, the marine luciferases and their engineered variants are highly thermostable, as indicated by their ability to retain bulk of their activity even after incubation at higher temperatures (≥48 °C)^7,11,19,20^, thereby allowing handling and storage of samples at room temperature or higher post-experiment or during transportation. In contrast, Fluc loses most of its activity after incubation at 30 °C^7^. Moreover, marine luciferases also retain their activity upon freezing and thawing. We have observed that the supernatants can be frozen and thawed at a later time for running the assay without compromising quality (unpublished observation). Finally, in contrast to inconsistent results obtained with Fluc-based reporter release assays in the published literature^10^, we have made more than 80 cell lines stably expressing Gluc and other luciferases described in this study in the cytosol, and have not come across any cell line in which the Matador assay did not work as expected.

The Matador assay has several additional advantages. Unlike Cr^51^ or florescent dye release assays, there is no need to preload the target cell on the day of the experiment or perform laborious procedures of washing to remove excess label. Further, the assay eliminates the need for laborious multistep assay formats involving separation of cells from supernatant, which may lead to both operational errors and inaccuracies in calculation of cytotoxicity. Once the target and effector cells are co-cultured, the whole assay is performed by a single step addition of assay reagent, thus allowing the possibility of assessing cell mediated cytotoxicity in high-throughput fashion. Although, we have not yet tested it, we speculate that in addition to measurement of cytotoxicity at multiple time points, the assay can be multiplexed with other assays, such as measurement of cytokine release from target cells. Target cells expressing marine luciferases can be prepared in advance and can be readily used when needed. Additionally, we have also demonstrated that the Matador assay is equally sensitive when luciferases are expressed by transient transfection. To expand the utility of the Matador assay for some hard-to-transfect cells, we have cloned all the luciferases in retro-and/or lentiviral vectors. This allows generation of different target cells stably expressing the luciferase of choice, which can be frozen for long term usage and enable sharing of these cells with other investigators. In addition, we have shown that the assay can be performed on primary cells in which the luciferase is expressed transiently. However, when working with primary cultures containing a mixture of cells, there is a possibility that different sub-populations of cells may have different susceptibility to transduction with viral vectors or express different levels of the reporter. If measurement of cell death of a specific sub-population in a polyclonal sample is desired, it is highly recommended to isolate the specific sub-population, for example using flow cytometry, before the performance of the assay.

Apart from a striking increase in luminescence in the cellular supernatants upon cell death, we also observed a slight increase in luminescence in the cell pellets of target cells (Fig. 4). This could be due to the fact that loss of cell membrane integrity results in increased penetration of CTZ into the dead and dying cells, where it reacts with any Gluc that has been trapped inside the cells and has not yet leaked out. Thus, the Matador assay is not only based on the release of Gluc (and other reporters) into the media due to loss of membrane integrity but also on the increased penetration of the substrate (or other essential components) required for the reporter activity into cells that have lost membrane integrity. This suggests that the assay may be further modified by targeting the reporters to different cellular compartments, such as to the cell membrane by adding a myristoylation signal^21^. Similarly, amino acid sequences that target the reporter proteins to other cellular compartments (e.g., nucleus, mitochondria, and golgi) have been described in the literature^22–24^ and can be used to develop assays that measure the integrity of different cellular compartments and components (e.g. mitochondrial membrane integrity).

The Matador assay has many potential applications in biomedical research and manufacturing of cellular therapy products. The assay may be used to screen candidate agents, such as a panel of antibodies or chimeric antigen receptors, for their ability to induce cellular cytotoxicity against a panel of cell lines to select the candidate with the highest on-target and the least off-target activity. Similarly, the assay can be used to monitor toxicity on normal healthy cells and tissues. The assay may also be used to screen for compounds that can modulate cellular cytotoxicity induced by antibodies, bispecific antibodies and cellular therapy products. Having an established potency assay is an FDA requirement for Biological License Application (BLA) for manufacturing of cellular therapy products. Based on its sensitivity, specificity, speed, and convenience, the Matador assay can be a useful potency assay at various steps involved in the manufacturing of cellular therapy products. The utility of the assay, however, is not limited to immunotherapeutics, and it can potentially have broad applicability in multiple areas such as screening for small molecule compounds, anticancer agents and environmental toxins. Studies are currently underway to adapt the assay for such applications.

## Materials and Methods

### Construction of retro-and lentiviral based luciferase expression vectors

Luciferase constructs used in this study were designed to lack a secretory signal. The nucleotide sequences encoding luciferases from *Gaussia princeps (*Gluc)^25^, Nanoluc engineered from *Oplophorus gracilorostris (*Nluc)^7^, *Metridia longa (*Mluc7)^19^, *Lucicutia Ovaliformis* (Loluc)^11^, *Plueromamma abdominalis* (Paluc1)^11^, and *Heterorhabdus tanneri* (Htluc1)^11^ was obtained from GenBank. Tluc16 engineered from the marine copepod *Metridia* family was obtained from ThermoFisher Scientific. All the luciferases were codon optimized and gene-fragments encoding the optimized sequences were synthesized by GeneArt^TM^ or Integrated DNA Technologies (IDT). The MLuc7 cDNA also carried M43L and M110L substitutions. The corresponding substitutions in GLuc have been previously shown to result in Glow type luminescence suitable for high throughput applications. The gene fragments were used as templates in PCR reactions using custom primers to amplify the corresponding DNAs, which were then cloned in different expression vectors using standard molecular biology techniques. In some cases, the fragments were cloned in-frame with nucleotide sequences encoding different epitope tags, such as FLAG, ×3FLAG, AcV5 and HA. Some of the constructs also co-expressed antibiotic resistance genes in frame with the cDNAs encoding the luciferases and separated from them by 2A ribosomal skip sequences. Essentially a similar strategy was used to clone Fluc and Rluc except the plasmids encoding the corresponding cDNAs were used as template in PCR reactions. The pLenti-Blast vector was derived from pLenti6v5gw_lacz vector (Invitrogen; ThermoFisher Scientific) by removal of the lacZ gene. pLenti-MP2 was a gift from Pantelis Tsoulfas (Addgene plasmid # 36097) and was used to generate the pLenti-EF1α lentiviral vector by replacement of the CMV promoter with human EF1α promoter using standard molecular biology techniques. psPAX2 was a gift from Didier Trono (Addgene plasmid # 12260). The pLP/VSVG envelope plasmid and 293FT cells were obtained from Invitrogen (ThermoFisher Scientific). The retroviral transfer vector MSCVneo, MSCVhygro, and MSCVpac and the packaging vector pKAT were obtained from Dr. Robert Illaria’s laboratory. phRGTK Renilla Luciferase plasmid was from Promega.

### Construction of lentiviral based chimeric antigen receptors (CARs)

To construct lentiviral vectors encoding FMC63-BBz Chimeric antigen receptor (CAR), the gene fragment encoding FMC63 CAR was synthesized by GeneArt^TM^ using the published sequence of FMC63 scFv^26^. The gene fragment was then used as template in a PCR reaction to amplify the sequence which was then cloned in to a lentiviral vector driven by an EF1α promoter and in frame with T2A ribosomal skip sequence followed by eGFP nucleotide sequence using standard molecular biology techniques. The resulting construct was labeled pLenti-EF1α-FMC63-MYC-BBz-T2A-eGFP. A lentiviral construct, pLenti-EF1α-FMC63-MYC-BBz-T2A-Pac was also constructed by replacement of eGFP gene with a puromycin resistance gene (Pac).

### Cell lines and reagents

K562 (Chronic Myelogenous Leukemia), Raji (Burkitt’s lymphoma), HEL 92.1.7 (Erythroleukemia) and NK92MI cell lines were obtained from ATCC and maintained as per the instructions provided. Nalm6 (Acute Lymphoblastic Leukemia), BC-1 (Primary effusion lymphoma), and KG-1 (Acute myelogenous leukemia) cell lines were kindly provided by Drs. Markus Muschen, Jae Jung, and Alan Epstein, respectively. 293FT cells were obtained from Invitrogen (ThermoFisher Scientific) and cultured as recommended. Peripheral blood mononuclear cells (PBMCs) were isolated by Ficoll gradient method from platelet depleted donor cells obtained from a Blood Bank. PBMCs were subsequently used to isolate T cells using CD3 magnetic microbeads (Miltenyi Biotech) following the manufacturer’s instructions. T cells were cultured in XVIVO-15 (Lonza) medium supplemented with 100 IU/ml recombinant human IL2 and 30 ng/ml soluble antibodies to human CD3 and CD28. All the cells were cultured at 37 °C, in a 5% CO2 humidified incubator. Blinatumomab was obtained from Amgen, Rituximab was obtained from Genentech. Digitonin, and Polybrene were from Sigma. Coelenterazine was purchased from Nanolight technology. Calcein AM fluorescent dye was obtained from BD biosciences.

### Preparation of retro-and lentivirus supernatants

Lentiviruses and retroviruses were generated in 293FT cells essentially as described previously^27^. For lentivirus production, 10 micrograms (μg) of lentiviral expression plasmid was used along with 7.5 μg of PSPAX2 and 2 μg of PLP/VSVG plasmid as packaging vector for a 100 mm plate (containing 10 ml media). Lentiviral titer was checked by measuring the amount of p24 antigen in concentrated viral supernatant. Essentially a similar procedure was used for production of retroviruses except 4 μg of pKAT and 2 μg of VSVG plasmids were used as packaging vectors.

### Transient transfection of 293FT cells with retro-and lentiviral based luciferase expression vectors

The 293FT cells were transfected in a 24-well plate with various expression plasmids (500 ng/well) using calcium phosphate as described previously^28^. Approximately, 18 hours post-transfection, the cells were either left untreated or treated with 30 μg/ml digitonin to induce cell death, followed by the detection of luminescence as described under luciferase assays.

### Generation of stable cancer cell lines expressing various luciferases

Stable cell lines expressing the different luciferase reporters were generated by infecting approximately 0.5 × 10^6^ cells in 1 ml of medium with 2 ml of viral supernatant in the presence of polybrene (8 μg/ml) in a 6-well plate by spin-infection (1800 rpm for 90 minutes at 37 °C). Next morning, the cells were pelleted and re-suspended in media with respective antibiotics for selection of stably transduced cells.

### Infection of primary human T-cells with chimeric antigen receptor encoding viruses

Second generation CAR constructs co-expressing a puromycin resistance gene were used to infect primary human T cells. The lentiviral supernatants were concentrated by centrifugation at 18,500 rpm for 2 hours at 4 °C. The viral pellets were re-suspended in 1/10 of the initial volume and stored at −80 °C. In general, primary T cells were infected using spin-infection as mentioned earlier, except 2 × 10^6^ cells/2 ml with 300 μl of concentrated virus in the presence of polybrene in morning. The media was changed in the evening and the infection was repeated for two more days for a total of 3 infections. After the 3^rd^ infection, the cells were pelleted and re-suspended in complete T-cell media with puromycin (400 ng/ml).

### Generation of NK92MI cells stably expressing CARs

NK92MI cells stably expressing second generation CAR constructs and coexpressing eGFP were generated by infecting with concentrated lentiviruses as described for primary human T cells. The eGFP positive cells were sorted using BD FACSAria II and expanded.

### Luciferase assays

Coelenterazine (CTZ) was used as a substrate to measure luminescence in cells expressing Gluc, Nluc, Mluc7, Tluc16, Paluc1, Htluc1, Loluc and Rluc using a CTZ-assay buffer containing 20 μM CTZ in PBS^29^. CTZ stock solution (2 mM) was made with acidified methanol as recommended by the manufacturer. Fluc activity was determined as described earlier^30,31^ by adding Fluc assay buffer (1 mM D-Luciferin, 25 mM Gly-Gly, 15 mM potassium phosphate, 15 mM MgSO_4,_ 4 mM EGTA, 2 mM ATP, and 1 mM DTT). Unless indicated otherwise, the assay buffer with substrate was added to cell culture media at 1:1 or at 1:4 ratio (v/v) for all luciferase assays. Luminescence was read in endpoint mode using BioTek synergy H4 hybrid microplate reader for 10 seconds. Percentage Specific lysis was calculated using the luciferase activity of digitonin-treated cells as maximum cell death and untreated cells as spontaneous cell death and using the formula % specific lysis = 100 × [(experimental data − spontaneous cell death)/(maximum cell death − spontaneous cell death)].

### LDH release assay

LDH release was measured using the CytoTox 96® Non-Radioactive assay kit (Promega). Briefly, cells were plated in the 96-well U bottom plate in phenol red-free medium and treated with cell death inducing agents or media (control). Post treatment, the plates were spun at 250 *g* for 4 minutes. Supernatants were carefully transferred into a flat bottom plate followed by the calorimetric detection of LDH activity as described in the manufacturer protocol.

### Calcein-release assay

Target cells were washed twice with phenol red free medium and re-suspended at 1 × 10^6^ per ml in medium containing 10 μM Calcein-AM, and incubated for 30 minutes at 37 °C. Post incubation, cells were washed twice with phenol red-free medium and plated in the 96-well U bottom plate, and treated with cell death inducing agents or media (control). Post treatment, the plates were spun at 250 *g* for 4 minutes, supernatants were carefully transferred to a black walled flat bottom plate and fluorescence (Excitation 485 ± 9 nm, Emission 530 ± 9 nm) was read in a Biotek Synergy Plate Reader.

### Culture and transduction of primary patient samples

De-identified primary patient samples were collected under an IRB approved protocol and frozen in liquid nitrogen. Cells were thawed and cultured overnight in RPMI containing 40% FBS, followed by removal of dead cells by Ficoll-Hypaque gradient centrifugation. 4 × 10^6^ cells were spin-infected with pLenti-Pac-Gluc at an MOI of 10 in the presence of polybrene (8 µg/mL). After 12–16 hours, cells were spun down and placed in fresh media. Cells were used for experiments 48 hours post infection. For CHTR-136, freshly isolated cells were used and cultured in primary T cell culture media (XVIVO-15 medium supplemented with 100 IU/ml recombinant human IL2, 30 ng/ml soluble antibodies to human CD3 and CD28).

### Fugene HD transfection

For transient transfection of K562 and Raji cells, Fugene HD transfection reagent (Promega) was used. After 48 hours, cells were plated at a density of 100 K cells/well in a white 384-well plate either alone or in the presence of the effector cells (NK92MI for K562 and FMC63-CAR-Ts for Raji) for 4 hours. The luminescence was measured as described earlier under luciferase assays.

### Statistical analysis

Two-tailed unpaired Student *t* test was used to test for differences between 2 groups using GraphPad Prism 5 software. Differences with a *P* ≤ 0.05 were considered statistically significant. All experiments were repeated a minimum of two times.

### Availability of data and reagents

The data and reagents will be available up on request to senior author P.M.C.

## Electronic supplementary material

Supplementary Information
