# Supplementary material for: Development and characterization of a novel luciferase based cytotoxicity assay
Source: Sci Rep. 2018 Jan 9;8:199. doi: 10.1038/s41598-017-18606-1 (PMC5760659; doi:10.1038/s41598-017-18606-1)
Supplement: Supplementary file 1 — Supplementary Information [file 41598_2017_18606_MOESM1_ESM.pdf]

SUPPLEMENTARY INFORMATION

Development and characterization of a novel luciferase based cytotoxicity assay

Hittu Matta*, Ramakrishnan Gopalakrishnan*, Sunju Choi*, Rekha Prakash, Venkatesh Natarajan, Ruben Prins, Songjie Gong, Saurabh D. Chitnis, Michael Kahn, Xu Han, Vishan Chaudhary, Adam Soni, Jennifer Se rnas, Prottasha Khan, Dan Wang and Preet M. Chaudhary#

*These authors contributed equally to this work.

Jane Anne Nohl Division of Hematology and Center for the Study of Blood Diseases, University of Southern California, Keck School of Medicine, Los Angeles, California, United States of America.

#**Corresponding author:** Preet M. Chaudhary, M.D., Ph.D.

Email: preet.chaudhary@med.usc.edu; **Phone:** 323-865-3916; Fax: 323-865-0060.

Supplementary Figure S1

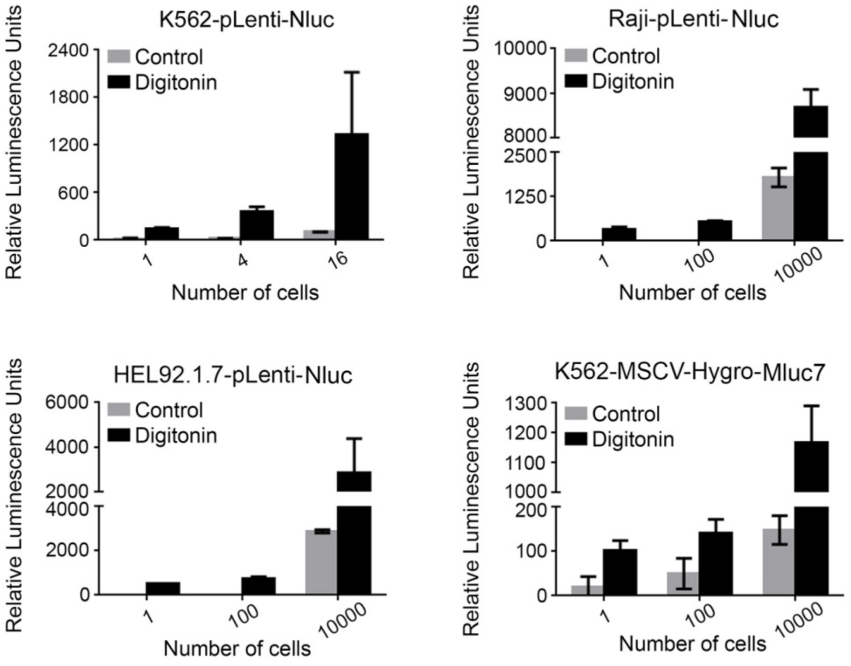

Supplementary Fig. S1. Matador assay is highly sensitive with linear increase in luminescence. Indicated cell lines stably expressing indicated luciferases were plated in a 384-well plate at indicated numbers (by serial dilution), and treated with digitonin (30µg/ml) for 90 minutes or vehicle (control). Luminescence was detected by the addition of coelenterazine containing assay buffer directly to each well, one at a time. The values shown are mean±SE of a representative experiment performed in triplicate for atleast two times.

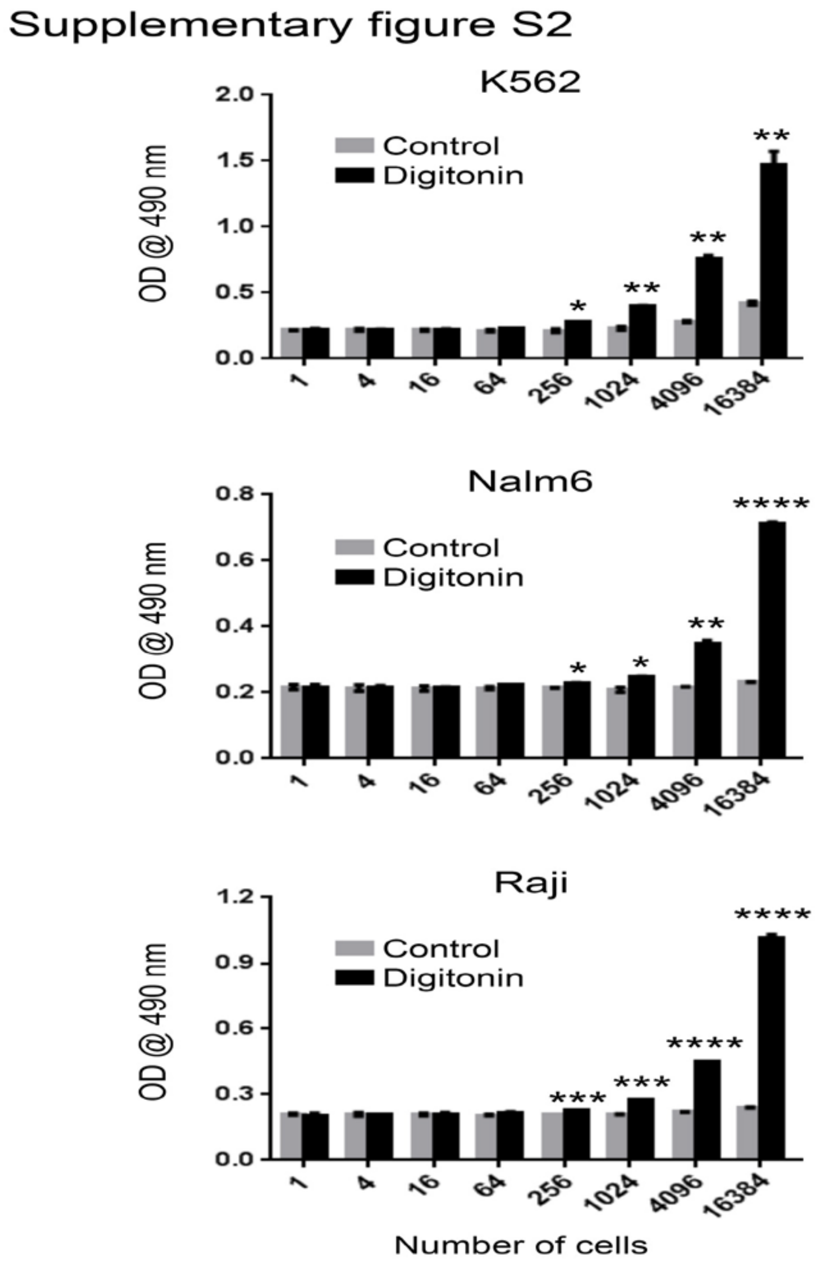

Supplementary Fig. S2. Sensitivity of LDH release assay. Indicated cell lines stably expressing pLenti-Pac-Gluc were plated in a 96-well U bottom plate in phenol red free media at indicated numbers (by serial dilution), and treated with digitonin (30µg/ml) for 120 minutes or vehicle (control). Post incubation the plates were spun at 250 x *g* for 4 minutes. Supernatants were carefully transferred into a flat bottom plate followed by the calorimetric detection of LDH activity as described in the manufacturer protocol (Promega CytoTox 96® Non-Radioactive assay kit). The values shown are mean±SE of a representative experiment performed in triplicate for atleast two times. Statistically significant differences were shown by asterisks (*) at a level of $P < 0.05$, (**) at a level of $P < 0.01$, (***) at a level of $P < 0.001$, and (****) at a level of $P < 0.0001$.

Supplementary figure S3

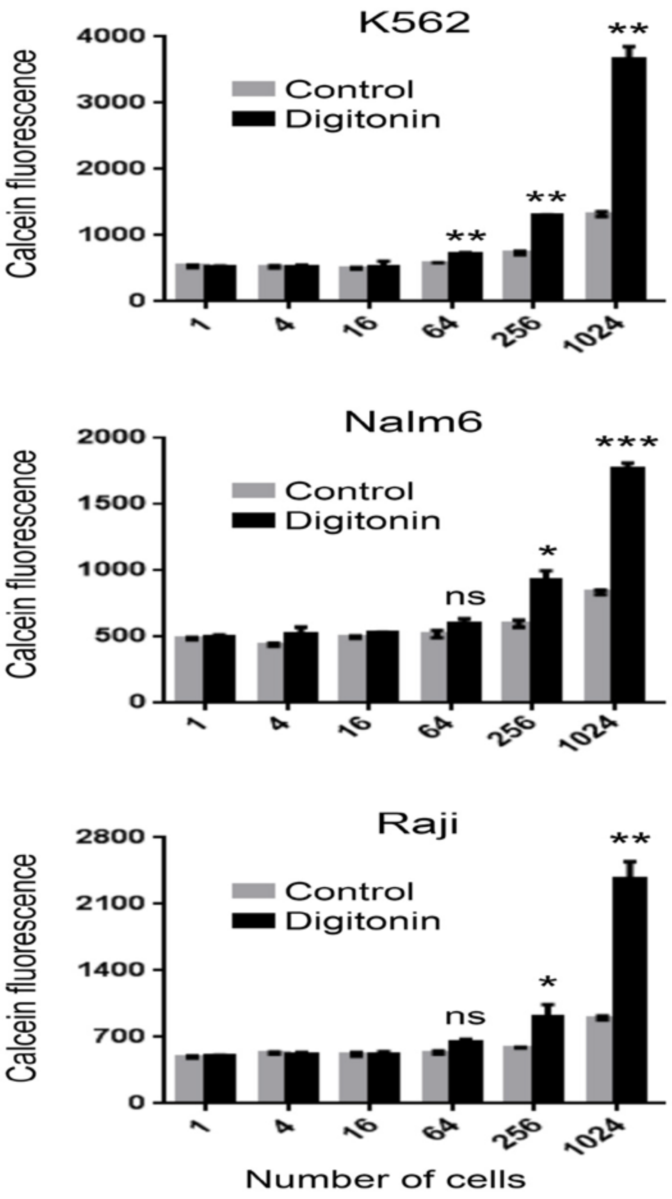

Supplementary Fig. S3. Sensitivity of Calcein release assay. Indicated cell lines stably expressing pLenti-Pac-Gluc were pre-incubated with Calcein-AM as described in methods section and were plated in a 96-well U bottom plate at indicated numbers (by serial dilution), and treated with digitonin (30µg/ml) for 120 minutes or vehicle (control). Calcein release was measured in the cleared supernatants by placing in a black walled flat bottom plate, followed by reading fluorescence (excitation 485±9nm, Emission 530± 9nm) in a biotek fluorescence plate reader. The values shown are mean±SE of a representative experiment performed in triplicate for atleast two times. Statistically significant differences were shown by asterisks (*) at a level of $P < 0.05$, (**) at a level of $P < 0.01$, and (***) at a level of $P < 0.001$. ns, not significant.

Supplementary figure S4

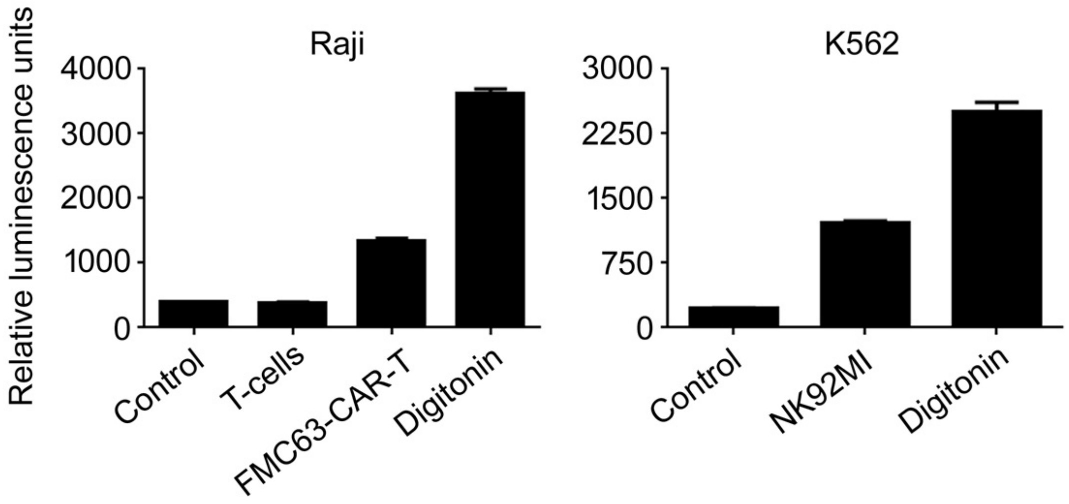

Supplementary Fig. S4. Matador assay works even at situations, when luciferase plasmids are transiently transfected in to cell lines. Raji and K562 cells were transfected with pLenti-Pac-Gluc plasmid DNA using Fugene HD (Promega). 48 hours post-transfection, Raji cells were co-incubated primary human T cells transduced FMC63-CAR at a E:T ratio of 10:1 for 4 hours. K562 cells were co-incubated with NK92MI at a E:T ratio of 0.5:1 for 4 hours. Gluc luminescence was measured as described in materials and methods. The values shown are mean±SE of a representative experiment performed in triplicate for atleast two times.
